# Solid Carbon Dioxide

**Published:** 1910-09-10

**Authors:** 


### Dermatology.

### SOLID CARBON DIOXIDE.

In the introduction of solid carbon dioxide as a cauterising agent general practitioners have obtained an important addition to their armamentarium. This substance fulfils all the conditions that can be reasonably asked of it in this capacity. It is efficacious, easy to use, cheap, readily obtained, and, in addition, possesses a quality which is not shared by any of the older cauterising agents, for it is painless. This quality, the importance of which it is difficult to exaggerate when dealing with nervous or very youthful patients, is due to the fact that the extreme cold of the cauterising pencil anæsthetises the sensory nerves. Solid carbon dioxide was not the first reagent to be used as a cautery in virtue of its freezing properties; that distinction is held by liquid air, which has been employed successfully for this purpose by Dr. Dade for more than seven years. The objections to this substance, however, are its greater cost, the difficulty of keeping it, and the more elaborate technique required. We owe, apparently, the introduction of solid carbon dioxide to Pusey of Chicago; one of the earliest papers on its employment is to be found in the *Dermatologishe Zeitschrift* for July 1908, by his colleague Zeisler.

The raw material is obtained in strong iron cylinders, in which it is confined under very high pressure. These cylinders, of course, are exactly the same as those in which oxygen is kept, and with which every medical man is familiar. They are to be obtained in several different sizes, either direct from the Carbon Dioxide Company or from any firm of dealers in motor-car accessories, for the gas is now used in increasing quantities to inflate motor tyres. It is much cheaper to buy cylinders of large size; but they are exceedingly heavy, and inconvenient to move about, so that for consulting-room work the smaller sizes are preferable. When confined in these cylinders the carbon dioxide is under very considerable pressure, and is therefore existing mostly in a liquid form, but some remain gaseous. Gases when they escape from very high pressure to a very low pressure lose large quantities of heat, and so when the carbon dioxide is allowed to escape from the cylinder into air it is cooled to such an extent that it freezes into a solid mass.

The only point in which any technical skill is required is in collecting it. This is best done by attaching to the nozzle of the cylinder a hollow pipe of some porous material. In practice this is most easily made by wrapping a stiff towel round a ruler and then withdrawing the ruler. The hollow cylinder thus formed is quite firm enough to retain its shape when fixed on to the nozzle by means of a bandage. The distal end of the towel may be with advantage partially closed by a cork. Nothing now remains but to turn on gently the tap. In a few moments the towel will be found full of a hard stick of solid carbon dioxide, which, on unwrapping the towel, is ready for use. Some care is necessary lest its freezing and cauterising properties be exercised upon the fingers of the operator. Either a piece may be chipped off with a pair of forceps and applied direct to the nævus or mole it is intended to destroy, or an elegant pencil may be made by placing some fragments in the bottom of a conical glass and stamping them together with a glass rod; or, again, the entire stick may be held in a towel while one end is carved into the required shape with a spatula.

The most suitable cases for the employment of carbon dioxide are nævi (especially the raised capillary variety), warts, moles, and the lesions of lupus erythematosus. Good results have also been recorded in cases of rodent ulcer, in which ulceration had not yet commenced, but the method is useless in cases at all advanced. In every instance the manner of application is the same. The prepared pencil is simply applied to the desired spot with a firm but gentle pressure. The most important point to be attended to is the duration of the application, for the effect produced is in direct proportion to the time during which the pencil is kept in contact with the skin. About 15 seconds is the shortest time in which it is possible to obtain a useful effect, and it is seldom advisable to allow a longer contact than about 40 seconds; it should, however, be mentioned that in dealing with luxuriant warts longer applications are necessary than with any other type of case. This no doubt is due to their toughness and their non-vascularity. Untoward results have been obtained by allowing too lengthy a contact; in fact, we have heard of a case of a nævus on the side of the nose in which necrosis took place to such a depth that the nasal cartilage was exposed, when the necrosed tissue separated. Those, therefore, who are employing solid carbon dioxide for the first time are advised to err, if at all, on the side of brevity.

The phenomena to be observed when all goes well may be shortly described as follows: On removing the pencil the area of its application is seen to be obviously depressed below the surrounding level and is dead white in colour; it is, in fact, of an appearance exactly similar to that produced by an ether spray, and if palpated found to be quite hard. The surrounding skin is unchanged in appearance. After a few moments, however, the whiteness disappears, and there is an almost instantaneous flow of lymph, which raises the previously depressed area above the normal level, and at the same time this area becomes surrounded by a zone of hyperæmia of some depth. This, the stage of reaction, lasts for some hours, and gradually subsides. In the course of a day or so superficial necrosis takes place, and a scab forms over the area of application, which separates in about a week or ten days. For the destruction of raised nævi one application is usually sufficient unless the nævus be a very extensive one; port-wine stains require more prolonged treatment, and so does lupus erythematosus. The scars left after this treatment compare favourably with those left after any other form of cautery. It is not recommended for lupus vulgaris.

